# Ultrasonographic Evaluation of Canine Hip Dysplasia: Comparison with FCI Radiographic Scoring System

**DOI:** 10.3390/vetsci13010020

**Published:** 2025-12-24

**Authors:** Inês Tomé, Sofia Alves-Pimenta, Bruno Colaço, Mário Ginja

**Affiliations:** 1 Department of Veterinary Sciences, University of Trás-os-Montes e Alto Douro, 5000-801 Vila Real, Portugal; inestome@utad.pt; 2 CECAV, Center for Animal Sciences and Veterinary Studies, University of Trás-os-Montes e Alto Douro, 5000-801 Vila Real, Portugal; salves@utad.pt (S.A.-P.); bcolaco@utad.pt (B.C.); 3 Department of Animal Science, University of Trás-os-Montes e Alto Douro, 5000-801 Vila Real, Portugal

**Keywords:** ultrasound, hip dysplasia, FCI score, diagnosis, dogs

## Abstract

Canine hip dysplasia (CHD) is a common orthopedic condition characterized by joint laxity, abnormal femoral head development, and osteoarthritis. Radiography remains the gold standard in diagnosis; however, ultrasonography (US) can detect changes in bone and periarticular soft tissue earlier in CHD progression. Forty-four hips from twenty-two adult dogs of various breeds were graded according to the Fédération Cynologique Internationale (FCI) grading system, and grouped as normal (A, B) or dysplastic (C, D, E). Canine hip US evaluation, using the ventral femoral head–neck approach, included the following: capsule thickness at the femoral head index (CTFHi) and capsule thickness at the femoral head–neck index (CTFHNi), both measured in mm/body weight × 100; femoral head shape score (FHSs) and femoral head–neck transition score (FHNTs); and osteophyte score (Os). These findings were evaluated qualitatively and then converted into numerical scores. Twenty-three hips were graded on the FCI system as being normal, and twenty-one as dysplastic. Median values of the US parameters CTFHi, CTFHNi, FHSs, FHNTs, and Os were 2.02, 7.79, 1.00, 1.00, and 0.00 in the normal-hips group, and 3.11, 9.32, 3.00, 2.00, and 1.00 in the dysplastic-hips group. Significant differences were observed between most US parameters evaluated. Strong associations were found between CTFHi, FHNTs, and Os, indicating progressive bone remodeling. These findings support US usage as a potential tool for CHD diagnosis and monitoring.

## Simple Summary

Canine hip dysplasia (CHD) is a very frequently occurring orthopedic disease in dogs which leads to pain, reduced mobility, and osteoarthritis. Radiography is the standard method for diagnosing and grading this disease, but this requires exposure to ionizing radiation and the use of deep sedation/anesthesia. In this study, we compared radiographic and ultrasonographic findings obtained from hip joints in dogs, and noted some specific ultrasound (US) signs. US detected thickening of the joint capsule, loss of femoral head shape, irregular head–neck transitions, and osteophytes, with changes more evident in dysplastic hip joints. These results may support the use of US as a potential tool in the diagnosis and monitoring of CHD.

## 1. Introduction

Canine hip dysplasia (CHD) is one of the most prevalent orthopedic diseases in dogs, particularly in medium- and large-breed populations [1,2,3]. It is a hereditary disease primarily associated with coxofemoral joint laxity, abnormal development of the femoral head, and progressive osteoarthritis [4,5,6,7]. This disease results in osteoarthritis, which is often followed by pain, functional limitations, and a poorer quality of life [8]. In veterinary medicine, radiography has been essential in CHD diagnosis for therapeutic and screening purposes [9,10]. Animals with radiographic signs of CHD are not recommended for breeding. This helps to reduce the occurrence of undesirable alleles in the canine population, and diminishes clinical and phenotypic manifestations of the disease [6,11].

Conventional radiographic assessment, especially using the ventrodorsal hip extended view, remains the gold standard for CHD diagnosis in adult animals [6]. The classification of CHD in adult animals by the Fédération Cynologique Internationale (FCI) scheme grades hips in five gradual categories from A (normal) to E (severely dysplastic), and is widely applied in most countries for clinical decision-making, breeding programs, and epidemiological studies [12,13].

Conventional radiography primarily detects advanced bony alterations, providing limited insight into early-stage CHD and changes in periarticular soft tissue [7,12,14,15]. Ultrasonography (US), on the other hand, provides a safe, ionizing-radiation-free imaging modality capable of providing dynamic images in real time without the need for deep sedation or general anesthesia [16,17,18]. It also identifies some osteoarthritic changes in bone, as well as changes in periarticular soft tissue and bone surface that may not be readily detected on radiographs [16,19,20]. Several studies have demonstrated the potential of US for identifying joint effusion, capsular thickening, osteophytes, and morphologic irregularities in the femoral head [19,20,21]. Capsular thickening has shown good sensitivity and specificity for distinguishing between normal and dysplastic hips, with increased thickness being strongly associated with increased hip joint laxity and osteoarthritic progression [19].

Recent methodological advances, including a standardized ventral hip approach to the femoral head–neck region, have enhanced the reproducibility and diagnostic value of US in veterinary orthopedics. This protocol allows detailed assessment of both the femoral head contour and the head–neck transition zone, providing valuable information on both early joint remodeling and established dysplastic changes [19]. Despite this growing evidence, quantitative ultrasonographic correlations with FCI grading remain limited. Therefore, this study aimed to associate the US parameters—capsule thickness at the femoral head index (CTFHi), capsule thickness at the femoral head–neck index (CTFHNi), femoral head shape score (FHSs), femoral head–neck transition score (FHNTs), and osteophyte score (Os)—with the FCI grading system. To the authors’ knowledge, no previous studies have investigated these hip US parameters; therefore, the premise that these parameters would not differ across FCI grades was established as the null hypothesis.

## 2. Materials and Methods

### 2.1. Animals

Twenty-two adult dogs of various breeds were presented for CHD screening at the Veterinary Hospital of the University of Trás-os-Montes and Alto Douro (UTAD) and were prospectively enrolled in the study with informed owner consent. Dogs with a history of hip joint trauma were excluded. All subjects underwent both radiographic and ultrasonographic examination of the hip joints. Breed, age, sex, and body weight were recorded. The sample size was estimated using a comparison table. A statistical significance of 0.05 and a large effect size of 0.8 were selected, along with a statistical power of 0.8, which indicated a minimum sample of 20 observations [22,23].

### 2.2. Imaging Assessment

Image acquisition was performed with the dogs under deep sedation; this was achieved using butorphanol (Butomidor^®^, Richter Pharma AG, Wels, Austria, at 0.2 mg/kg), dexmedetomidine (Sedadex^®^, Le Vet Beheer B.V., Oudewater, The Netherlands, at 1 μg/kg), and propofol (Propofol Lipuro^®^, B.Braun, Lisbon, Portugal, at 4 mg/kg) intravenously.

#### 2.2.1. Radiographic Assessment

Standard ventrodorsal hip-extended radiographs were taken (Optimus 80, Philips, Amsterdam, The Netherlands), and hips were evaluated according to the FCI grading system (grades A to E). For statistical analysis, hips were grouped as follows [12,13]:

- **Normal**-**Hips Group:** A and B FCI scoring. Normal hips (A) are characterized by a congruent femoral head and acetabulum, a sharp and rounded craniolateral acetabular rim, and a Norberg angle of around 105°. In near-normal hips (B), the acetabulum and femoral head may be slightly incongruent, and the Norberg angle is close to 105°.
- **Dysplastic-Hips Group:** C, D, and E FCI scoring. Mildly dysplastic hips (C) have an incongruent femoral head and acetabulum, a Norberg angle of around 100°, and may evidence slight osteoarthritic signs on the acetabular edge as well as flattening of the craniolateral acetabular rim. Moderately dysplastic hips (D) have clear incongruity between the femoral head and the acetabulum, subluxation, a Norberg angle of around 90°, and flattening of the craniolateral rim, or the presence of osteoarthritic signs. Severely dysplastic hips (E) have a more marked subluxation or even luxation, a Norberg angle less than 90°, obvious flattening of the cranial acetabular edge, and a mushroom-shaped/flattened femoral head, and they may exhibit other signs of osteoarthrosis.

The radiographic views were performed and analyzed by a single experienced examiner (MG).

#### 2.2.2. Ultrasonographic Examination

**Figure 1:**
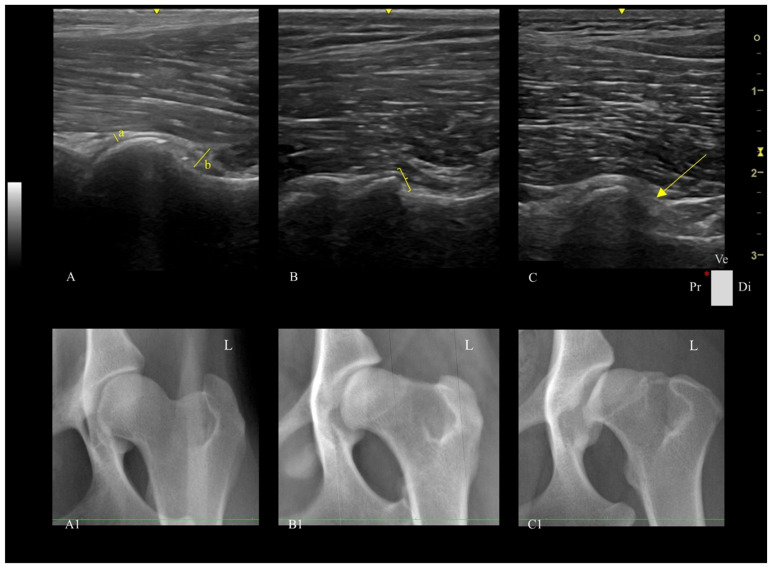
Ultrasound parameters were obtained from the longitudinal plane of the femoral head–neck plane in dogs. (**A**): Hip with a capsule thickness at the femoral head of 2.11 mm, measured at the maximum perpendicular distance of the joint capsule (a); a capsule thickness at the femoral head–neck index of 8.32 mm, measured as the perpendicular distance between the outermost and innermost layer of the joint capsule (b); with a spherical femoral head and a smooth head–neck transition score, without osteophytes. (**A1**): Corresponding X-ray of US image A. Hip presents a good femoral head–acetabulum congruity, a Norberg angle of approximately 105° and an A score, according to the FCI grading system. (**B**): Hip with a flattened femoral head and an irregular head–neck transition score (parentheses), without osteophytes. (**B1**): Corresponding X-ray of US image B. Hip presents moderate femoral head–acetabulum incongruity, slight signs of osteoarthritis of the cranial and dorsal acetabular edge and the femoral head and neck. A Morgan line is present. The Norberg angle is about 100°, equivalent to the FCI grade C. (**C**): Hip with a severely flattened femoral head and a highly irregular head–neck transition score, with the presence of osteophytes (arrow). (**C1**): Corresponding X-ray of US image C, showing severe femoral head–acetabulum incongruity, severe osteoarthritis signs, a flattened cranial acetabular edge, deformation of the femoral head, and a prominent Morgan line. The Norberg angle is inferior to 90 °C, corresponding to the FCI grade E. Ve—Ventral, Pr—Proximal, Di—Distal L—Left, * Probe orientation and indication marker.

Ultrasound assessment was performed using a portable US machine (Logiq e, General Electric Medical Systems, Buc, France) equipped with a high-frequency linear probe (L8-18i-RS, General Electric Medical Systems, Buc, France) in a standardized ventral longitudinal approach to the femoral head–neck region, as described by Tomé et al. [16]. The following parameters were assessed for each hip (Figure 1):

**1. Capsule thickness at the femoral head index (CTFHi):** measured in mm/body weight × 100. The capsule was measured as the maximum perpendicular distance between the outer and inner limits of the joint capsule.

**2. Capsule thickness at the femoral head–neck index (CTFHNi):** measured in mm/body weight × 100. This is the perpendicular distance between the outermost and innermost layers of the joint capsule, *stratum fibrosum* and *stratum synoviale*, respectively.

**3. Femoral head shape score (FHSs):** spherical, flattened, or severely flattened, represented by numerical values of 1, 2, and 3, respectively.

**4. Femoral head–neck transition score (FHNTs):** smooth, irregular, or highly irregular, represented by numerical values of 1, 2, and 3, respectively.

**5. Osteophyte score (Os):** absent or present, represented by numerical values of 0 and 1, respectively.

All US parameters were performed by a single experienced operator blinded to FCI grading (IT) using the Radiant Dicom Viewer (Medixant, Poznań, Poland).

### 2.3. Statistical Analysis

Data was analyzed using SPSS (IBM Statistics for Windows, Version 27.0, Armonk, NY, USA). Basic US features of the data were presented as continuous variables using mean, standard deviation, median, and interquartile range. Data were tested for normality using the Shapiro–Wilk test, and group differences were assessed using the Mann–Whitney U test for non-parametric variables. Also, a correlation analysis using Spearman’s coefficients was employed to evaluate the relationships between measured parameters. Effect size and post hoc power values were calculated for variables showing significant group differences [22,23]. A *p* value < 0.05 was considered statistically significant.

## 3. Results

The mean body weight of dogs was 29.52 ± 14.93 kg (mean ± SD). Six different breeds were considered in this study, namely, seven Portuguese Sheepdogs, six Estrela Mountain dogs, three Transmontano Mastiff dogs, three Portuguese Pointer dogs, and three Border Collie dogs. Dogs were aged between 13 to 136 months, with a mean age of 41.00 ± 29.51 months.

**Figure 2:**
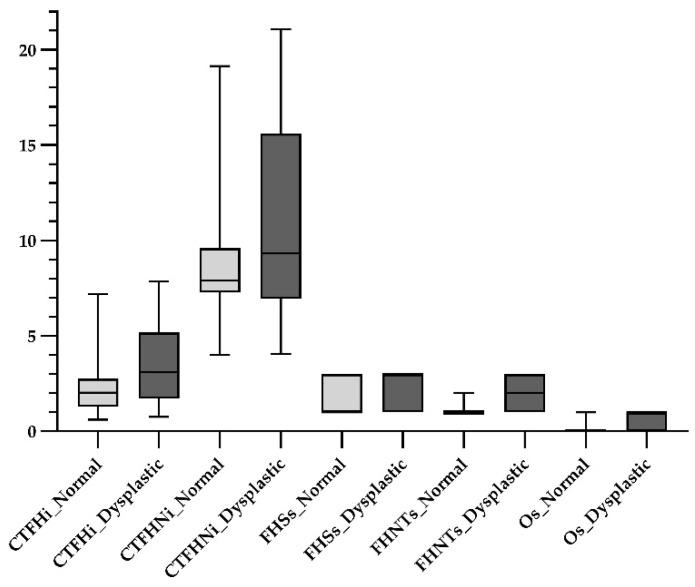
Boxplot graph illustrating the distribution of ultrasonographic parameters in the different radiographic assessment groups, with parameter values obtained from 44 hips. The evaluated parameters include capsule thickness at the femoral head index (CTFHi), capsule thickness at the femoral head–neck index (CTFHNi), femoral head shape score (FHSs), femoral head–neck transition score (FHNTs), and osteophyte score (Os). Light grey boxes represent the normal-hips group and the dark grey boxes the dysplastic-hips group. Each box represents the interquartile range (IQR) between the 25th and 75th percentiles, the horizontal line inside the box indicates the median, and the whiskers extend to the minimum and maximum values within 1.5× the IQR.

A total of 44 hips were evaluated radiographically and distributed according to the FCI grading system as follows: A normal-hips group with 23 hips (8 hips with FCI score A and 15 hips with FCI score B); and a dysplastic-hips group with 21 hips (6 hips with FCI score C, 11 hips with FCI score D, and 4 hips with FCI score E). These hips were then submitted to a US assessment following the established US-guided protocol [16], and different US parameters were collected from the images recorded. The median values of the US parameters CTFHi, CTFHNi, FHSs, FHNTs, and Os were, respectively, 2.02, 7.79, 1.00, 1.00, and 0.00 in the normal-hips group, and 3.11, 9.32, 3.00, 2.00, and 1.00 in the dysplastic hips-group (Figure 2). Statistically significant differences were observed in the US parameters CTFHi, FHSs, FHNTs, and Os between the normal- and dysplastic-hips groups, with a statistical power superior to 0.8, except for the CTFHi parameter [22,23] (Table 1).

Using Spearman correlation analysis, a very strong relationship was found between FHNTs and osteophyte scores; a strong relationship between CTFHNi and both FHNTs and Os scores; and a moderate relationship between CTFHi and CTFHNi, FHNTs, and Os scores. Other relationships between US parameters involved weak or low correlations (Table 2).

## 4. Discussion

This study demonstrated that ultrasonographic assessment of the canine hip joint, using a standardized longitudinal femoral head–neck approach, reveals measurable differences in soft tissue and bony structures among hips grouped according to FCI radiographic scoring in terms of the following different US parameters evaluated: CTFHi, FHSs, FHNTs, and Os.

Joint capsule thickening, especially over the femoral head, showed a significant trend across research groups, with higher values observed in the dysplastic group. Our findings are in agreement with previous studies, indicating that capsular thickening may reflect chronic synovial inflammation or joint effusion in response to hip laxity and instability [16,20,21]. Our results support the idea that the CTFHi, particularly when values were normalized to body weight, may act as a marker for CHD monitoring. Additionally, dogs in the dysplastic-hips group exhibited higher median values in CTFHNi, compared to the normal-hips group. Although this tendency was not statistically significant, it may reflect limited sensitivity to detect early structural variation or insufficient sample homogeneity rather than a true absence of association. Enhancements in the acquisition of US parameters may help further characterize and stratify the US research groups.

Importantly, alterations in FHSs and FHNTs also demonstrated statistically significant differences between groups. In the normal-hips group, the femoral head was consistently spherical with a smooth transition to the femoral neck. In contrast, the dysplastic-hips group displayed flattened femoral heads and highly irregular head–neck transitions in the US. These changes reflect biomechanical adaptations to chronic subluxation and altered joint loading, important hallmarks of progressive CHD [12,24,25,26]. Our results support these findings; the dysplastic-hips group showed significantly more severe morphological alterations than the normal-hips group. The transition from smooth to irregular contours at the femoral head–neck junction has been previously highlighted as an early sign of joint remodeling and as a predictor of osteoarthritic changes [16].

The Os was revealed to be the US assessment feature with the strongest associations within the different FCI classification groups. The dysplastic-hips group demonstrated a significantly higher frequency of osteophyte formation than the normal-hips group, with the latter group having a frequency of zero. This result confirms that the FCI grades A and B exhibit a lower probability of developing osteophytes compared to grades C, D, and E, where its development is more common. Osteophyte formation is a hallmark of late-stage joint degeneration and a major determinant in radiographic CHD grading [27,28]. Our results may confirm that US can sensitively detect osteophytes even in the early stages, reinforcing its utility for longitudinal monitoring and early diagnosis of CHD.

The Spearman correlation analysis provided additional insights into the relationships between ultrasonographic parameters. Strong positive correlations were found between CTFHNi and both FHNTs and Os, showing that changes in soft tissue and bone progress as hip dysplasia worsens. This is in line with previous studies where capsular thickening and periarticular bone changes were described as secondary responses to joint laxity and instability [16,20]. The FHNTs and Os were very strongly associated, consistent with other reports highlighting that irregular bone contours predispose to osteophyte formation as part of the degenerative cascade [16]. CTFHi also correlated moderately with CTFHNi, FHNTs, and Os, suggesting that it can serve as an early indicator of joint stress and joint remodeling. This finding is sustained by the work of Souza et al. [20], who demonstrated associations between capsule thickness, elastography changes, and radiographic grading. The FHSs showed weaker correlations, which may reflect the fact that loss of sphericity is a more gradual morphological alteration, occurring later than capsular or bone proliferative changes [13]. Overall, these results suggest not only that US parameters are useful in categorical comparisons, but also that they alter proportionally with disease severity and progression, highlighting the novelty and clinical relevance of the present study. Unlike previous studies that focused primarily on joint capsule thickness or qualitative descriptions, this study integrates quantitative US metrics for soft tissue and bone with FCI grading, thereby establishing reproducible imaging biomarkers for CHD classification. These parameters can support early screening and follow-up without relying on ionizing radiation.

In the present study, the standardized longitudinal femoral head–neck plane in a ventral approach to the ventral hip joint was chosen because it has been shown to provide consistent sonoanatomic landmarks as well as high reproducibility in evaluating joint capsule thickness, femoral head contour, and head–neck transition morphology [16]. This plane also minimizes acoustic shadowing from the pelvis and allows direct assessment of periarticular remodeling, making it particularly suited for studies of adult dogs in which chronic changes predominate. In contrast, the transverse femoral head–neck plane is mainly recommended for detecting increased synovial volume and joint recess changes, which are early indicators of joint instability and inflammation [16,18]. These features are especially valuable in young dogs during the initial stages of CHD, when effusion may be present before structural remodeling is evident [16]. However, in adult dogs, in which the disease has typically progressed to include capsular thickening, femoral head deformation, and femoral head–neck transition type and osteophyte development, such findings are more discriminative. For this reason, we focused on the longitudinal plane, as it provides more relevant information in the context of chronic CHD changes, while acknowledging that transverse and cranial recess assessments remain important for the early diagnosis of CHD in younger animals. Moreover, this study provides quantitative evidence that a standardized longitudinal femoral head–neck approach captures both periarticular soft tissue changes (CTFHi, CTFHNi) and morphological bone alterations (FHSs, FHNTs, Os) that correlate with FCI radiographic grade, reinforcing US role as a multimodal tool for CHD assessment beyond simple detection of luxation [17].

Breed variability is a common limitation in CHD studies due to differences in joint conformation and soft tissue proportions. By normalizing joint capsule thickness using body weight, we attempted to mitigate breed- and size-related measurement variability, which may enable better inter-breed comparisons and improve the reproducibility of results [7,29,30]. Although the power analysis indicated sufficient sensitivity for large effects, future studies with larger breed-specific cohorts are warranted to refine US thresholds and validate interbreed applicability.

This study presents several limitations. The relatively small and heterogeneous sample, despite adequate power for the main comparisons, limits breed-specific generalization. The exploratory methodological design of this study aimed to establish feasible ultrasonographic reference parameters for future population-level studies. Moreover, the consistency of significant differences across related US variables supports the adequacy of the sample for the intended purpose. Additionally, inter- and intraobserver variability testing was not assessed. Nevertheless, all ultrasonographic examinations were performed by a single experienced operator using a previous standardized protocol, minimizing procedural variability and ensuring consistency. Future research including multiple observers and larger breed-homogeneous populations is warranted to validate these findings.

Lastly, this study focused on a single standardized ultrasonographic view. While this ensured consistency and practical applicability, it may have failed to detect some changes in soft tissue and bone structure that might have been visible with other US approaches. Future studies should explore multimodal US planes, interobserver reliability, and longitudinal assessment of dogs as they progress through dysplastic changes. Despite these limitations, the present study contributes new quantitative evidence supporting the diagnostic potential of US for CHD, especially in identifying early structural changes prior to radiographic alterations. By integrating US into CHD screening protocols, veterinarians may reduce radiation exposure, facilitate earlier therapeutic decisions, and improve welfare outcomes for predisposed breeds.

## 5. Conclusions

Ultrasound of canine hip joints using the standardized longitudinal femoral head–neck approach detected imaging-relevant differences in CTFHi, FHSs, FHNTs, and Os between dogs with different degrees of radiographic CHD, according to the FCI grading scheme. Correlations were found between the US parameters assessed. These findings may support the use of US as a potential screening tool to enhance the assessment of CHD, highlighting the clinical relevance of this study.

## Figures and Tables

**Table 1:** Descriptive statistics reported as median ($\tilde{x}$) and interquartile range (IQR), along with values for effect size and post hoc statistical power for ultrasonographic parameters across the radiographic assessment groups (n = 44 hips).

US Parameters	Normal Hips (n = 23)		Dysplastic Hips (n = 21)		Size Effect	Power
	$\tilde{X}$	IQR	$\tilde{X}$	IQR		
Capsule Thickness at the Femoral Head Index	2.02 ^a^	1.61	3.11 ^b^	3.46	0.62	<0.80
Capsule Thickness at the Femoral Head–Neck Index	7.79 ^a^	1.93	9.32 ^a^	8.64	_	_
Femoral Head Shape Score	1.00 ^a^	1.50	3.00 ^b^	2.00	0.79	>0.80
Femoral Head–Neck Transition Score	1.00 ^a^	0.00	2.00 ^b^	2.00	1.18	>0.80
Osteophyte Score	0.00 ^a^	0.00	1.00 ^b^	1.00	1.14	>0.80

US parameter median values for research groups with different superscript letters are statistically significantly different (*p* < 0.05) in Mann–Whitney U testing.

**Table 2:** Spearman correlations among ultrasonographic parameters obtained from adult dogs (n = 44 hips).

Ultrasound Parameters	Capsule Thickness at the Femoral Head Index	Capsule Thickness at the Femoral Head–Neck Index	Femoral Head Shape Score	Femoral Head–Neck Transition Score	Osteophyte Score
Capsule Thickness at the Femoral Head Index	1	0.59 *	0.09	0.57 *	0.58 *
Capsule Thickness at the Femoral Head–Neck Index		1	0.24	0.70 *	0.69 *
Femoral Head Shape Score			1	0.37 *	0.36 *
Femoral Head–Neck Transition Score				1	0.99 *
Osteophyte Score					1

* indicates a statistically significant correlation between the variables.

## Data Availability

Data is contained within the article.

## Abbreviations

The following abbreviations are used in this manuscript:

CHD	Canine Hip Dysplasia
US	Ultrasonography
FCI	Fédération Cynologique Internationale
CTFHi	Capsule Thickness at the Femoral Head Index
CTFHNi	Capsule Thickness at the Femoral Head–Neck Index
FHSs	Femoral Head Shape Score
FHNTs	Femoral Head–Neck Transition Score
Os	Osteophyte Score
UTAD	University of Trás-os-Montes and Alto Douro

## References

[1] Willemsen K., Möring M.M., Harlianto N.I., Tryfonidou M.A., van der Wal B.C.H., Weinans H., Meij B.P., Sakkers R.J.B. (2021). Comparing Hip Dysplasia in Dogs and Humans: A Review. Front. Vet. Sci..

[2] Roberts T., McGreevy P.D. (2010). Selection for Breed-Specific Long-Bodied Phenotypes Is Associated with Increased Expression of Canine Hip Dysplasia. Vet. J..

[3] Ohlerth S., Geiser B., Flückiger M., Geissbühler U. (2019). Prevalence of Canine Hip Dysplasia in Switzerland Between 1995 and 2016—A Retrospective Study in 5 Common Large Breeds. Front. Vet. Sci..

[4] Butler J., Gambino J. (2017). Canine Hip Dysplasia: Diagnostic Imaging. Vet. Clin. Small Anim. Pract..

[5] Mikkola L., Holopainen S., Pessa-Morikawa T., Lappalainen A., Hytönen M., Lohi H., Iivanainen A. (2019). Genetic dissection of canine hip dysplasia phenotypes and osteoarthritis reveals three novel loci. BMC Genom..

[6] Ginja M.M.D., Silvestre A.M., Gonzalo-Orden J.M., Ferreira A.J.A. (2010). Diagnosis, Genetic Control and Preventive Management of Canine Hip Dysplasia: A Review. Vet. J..

[7] Smith G.K., Biery D.N., Gregor T.P. (1990). New Concepts of Coxofemoral Joint Stability and the Development of a Clinical Stress-Radiographic Method for Quantitating Hip Joint Laxity in the Dog. J. Am. Vet. Med. Assoc..

[8] Osteoarthritis: A Serious Disease. https://oarsi.org/oarsi-white-paper-oa-serious-disease.

[9] Dennis R. (2012). Interpretation and Use of BVA/KC Hip Scores in Dogs. Clin. Pract..

[10] Reagan J. (2017). Canine Hip Dysplasia Screening Within the United States: Pennsylvania Hip Improvement Program and Orthopedic Foundation for Animals Hip/Elbow Database. Vet. Clin. Small Anim. Pract..

[11] Soo M., Worth A.J. (2015). Canine Hip Dysplasia: Phenotypic Scoring and the Role of Estimated Breeding Value Analysis. N. Z. Vet. J..

[12] Flückiger M. (2007). Scoring Radiographs for Canine Hip Dysplasia–The Big Three Organisations in the World. Eur. J. Companion Anim. Pract..

[13] Pinna S., Tassani C., Antonino A., Vezzoni A. (2022). Prevalence of Primary Radiographic Signs of Hip Dysplasia in Dogs. Animals.

[14] Carrig C.B. (1997). Diagnostic Imaging of Osteoarthritis. Vet. Clin. Small Anim. Pract..

[15] Roemer F.W., Guermazi A., Demehri S., Wirth W., Kijowski R. (2022). Imaging in Osteoarthritis. Osteoarthr. Cart..

[16] Tomé I., Alves-Pimenta S., Colaço B., Ginja M. (2025). Ultrasonographic Ventral Hip Joint Approach and Relationship with Joint Laxity in Estrela Mountain Dogs. Animals.

[17] Todd-Donato A.B., VanDeventer G.M., Porter I.R., Krotscheck U. (2024). Ultrasound Is an Accurate Imaging Modality for Diagnosing Hip Luxation in Dogs Presenting with Hind Limb Lameness. J. Am. Vet. Med. Assoc..

[18] Sudula S. (2016). Imaging the Hip Joint in Osteoarthritis: A Place for Ultrasound?. Ultrasound.

[19] Tomé I., Alves-Pimenta S., Costa L., Pereira J., Sargo R., Brancal H., Ginja G., Colaço B. (2023). Establishment of an Ultrasound-Guided Protocol for the Assessment of Hip Joint Osteoarthritis in Rabbits—A Sonoanatomic Study. PLoS ONE.

[20] Carneiro R.K., da Cruz I.C.K., Gasser B., Lima B., Aires L.P.N., Ferreira M.P., Uscategui R.A.R., Giglio R.F., Minto B.W., Rossi Feliciano M.A. (2023). B-Mode Ultrasonography and ARFI Elastography of Articular and Peri-Articular Structures of the Hip Joint in Non-Dysplastic and Dysplastic Dogs as Confirmed by Radiographic Examination. BMC Vet. Res..

[21] Madsen J.S. (1997). The Joint Capsule and Joint Laxity in Dogs with Hip Dysplasia. J. Am. Vet. Med. Assoc..

[22] Cohen J. (1988). Statistical Power Analysis for the Behavioral Sciences.

[23] How2statsbook. https://www.how2statsbook.com/.

[24] Tomé I., Alves-Pimenta S., Sargo R., Pereira J., Colaço B., Brancal H., Costa L., Ginja M. (2023). Mechanical Osteoarthritis of the Hip in a One Medicine Concept: A Narrative Review. BMC Vet. Res..

[25] Prieur W.D. (1980). Coxarthrosis in the Dog Part I: Normal and Abnormal Biomechanics of the Hip Joint. Vet. Surg..

[26] Weigel J.P., Wasserman J.F. (1992). Biomechanics of the Normal and Abnormal Hip Joint. Vet. Clin. Small Anim. Pract..

[27] Vandevelde B., Van Ryssen B., Saunders J.H., Kramer M., Van Bree H. (2006). Comparison of the ultrasonographic appearance of osteochondrosis lesions in the canine shoulder with radiography, arthrography, and arthroscopy. Vet. Radiol. Ultrasound.

[28] De Lasalle J., Alexander K., Olive J., Laverty S. (2016). Comparisons among radiography, ultrasonography and computed tomography for ex vivo characterization of stifle osteoarthritis in the horse. Vet. Radiol. Ultrasound.

[29] Brønniche M., Pedersen T., Mouritzen A., Vitger D., Nielsen N., Poulsen H., Miles E. (2020). Kinetic gait analysis in healthy dogs and dogs with osteoarthritis: An evaluation of precision and overlap performance of a pressure-sensitive walkway and the use of symmetry indices. PLoS ONE.

[30] Tuchpramuk P., Kaenkangploo D., Srithunyarat T., Seesupa S., Hoisang S., Lascelles X., Kampa N. (2025). Force Plate Gait Analysis in Dogs After Femoral Head and Neck Excision. Vet. Sci..

